# Transcatheter arterial perfusion chemotherapy combined with lipiodol chemoembolization for advanced colorectal cancer complicated by obstruction

**DOI:** 10.3389/fonc.2024.1369829

**Published:** 2024-04-26

**Authors:** Xiaolong Ding, Yaozhen Ma, Meipan Yin, Tao Liu, Shuiling Jin, Chunxia Li, Xiaobing Li, Chenchen Zhang, Gang Zhou, Gang Wu

**Affiliations:** ^1^ Department of Interventional Radiology, The First Affiliated Hospital of Zhengzhou University, Zhengzhou, China; ^2^ Department of Gastrointestinal Surgery, The First Affiliated Hospital of Zhengzhou University, Zhengzhou, China; ^3^ Department of Oncology, The First Affiliated Hospital of Zhengzhou University, Zhengzhou, China

**Keywords:** colorectal cancer, obstruction, lipiodol, chemoembolization, interventional radiology

## Abstract

**Background:**

Obstruction is a common complication of advanced colorectal cancer. This study was aimed at investigating the safety, efficacy, and feasibility of transcatheter arterial perfusion chemotherapy combined with lipiodol chemoembolization for treating advanced colorectal cancer complicated by obstruction.

**Patients and methods:**

This retrospective analysis was conducted using clinical data of patients with advanced colorectal cancer who received arterial infusion chemotherapy combined with lipiodol chemoembolization treatment at our center. Treatment efficacy was evaluated in terms of obstruction-free survival and overall survival, and treatment complications were monitored.

**Results:**

Fifty-four patients with colorectal cancer complicated by obstruction were included. All patients successfully underwent transcatheter arterial infusion combined with lipiodol chemoembolization treatment. The average lipiodol dose administered was 2.62 ± 1.45 ml (0.5–5.5 ml). No serious complications such as perforation or tumor dissemination occurred. The clinical success rate was 83.3% (45/54). One month after treatment, the objective response rate (ORR) and disease control rate (DCR) were 66.67% and 88.9%, respectively. The median obstruction-free survival was 5.0 months. No serious adverse events occurred. As of the last follow-up, 6 patients survived, 44 died, and 4 were lost to follow-up.

**Conclusion:**

Our findings revealed that transcatheter arterial infusion chemotherapy combined with lipiodol chemoembolization is safe and effective for treating advanced colorectal cancer complicated by obstruction. It may serve as a new treatment strategy for patients with advanced colorectal cancer complicated by obstruction.

## Introduction

Colorectal cancer (CRC) is one of the most common malignant tumors of the digestive system in clinical settings and ranks third among malignant tumors in terms of incidence and mortality (1). Surgery is the preferred method for non-advanced colorectal cancer. However, 40-50% of patients with CRC already have distant metastasis at the time of diagnosis, precluding safe surgery. The overall 5-year survival rate in these patients is less than 10% (2, 3).

Malignant obstruction is a common complication of advanced CRC and is noted in more than 30% of such patients (4, 5). If the obstruction is not resolved promptly, it can lead to a continuous increase in intestinal pressure, thinning of the intestinal wall, and obstruction of venous reflux. In severe cases, intestinal ischemia and necrosis develop, leading to rapid deterioration of patients’ condition. Although most surgical procedures can quickly relieve obstruction, the incidence of surgical complications and postoperative mortality are relatively high owing to factors such as poor tolerance and intestinal edema in patients with advanced CRC (6, 7). Although the use of self-expanding metal stents (SEMS) can quickly relieve obstruction, the treatment has significant limitations, and with tumor progression, obstruction is prone to recurrence (8). Transcatheter arterial chemoembolization (TACE) is an effective treatment for the management of liver metastasis of CRC (9). However, no studies have investigated the use of TACE for treating CRC complicated by obstruction. TACE blocks the tumor-supplying artery and tumor vascular bed, continuously releases chemotherapy drugs, quickly reduces tumor volume, and relieves intestinal obstruction symptoms. Transcatheter arterial infusion (TAI) involves direct injection of chemotherapy drugs into the tumor-nourishing artery and is characterized by a high local drug concentration and precise therapeutic effect. The method involves the use of low doses of chemotherapy drugs; any resulting toxicity and side effects are also of low severity (10). It has shown good therapeutic effects in the fields of liver and lung cancers (11, 12). Hence, this study was aimed at evaluating the safety, effectiveness, and feasibility of TAI combined with lipiodol chemoembolization (TACE) in the treatment of advanced CRC complicated by obstruction.

## Patients and methods

### Patient data

Clinical data of patients with advanced CRC complicated by obstruction and who received TAI combined with lipiodol chemoembolization treatment at the First Affiliated Hospital of Zhengzhou University from August 2013 to April 2023 were retrospectively analyzed. This included progress notes, imaging data, laboratory results, surgical procedure records, and follow-up results. Inclusion criteria were as follows: 1) a clear diagnosis of CRC through imaging, endoscopy, and pathologic tests; 2) confirmed presence of intestinal obstruction through clinical symptoms and imaging studies; and 3) surgical treatment judged as not feasible after evaluation by a gastroenterologist or patient’s refusal to undergo surgery. The following patients were excluded: 1) patients who simultaneously received any other antitumor treatment; 2) patients with obstruction caused by non-primary CRC or postoperative intestinal adhesions; 3) patients showing disease progression to intestinal perforation and rupture; and 4) patients who underwent implantation of SMES implantation or fistula surgery. All patients and their families signed an informed consent form. The study was approved by the Ethics Committee of the First Affiliated Hospital of Zhengzhou University (2021-KY-0911-004).

### Preoperative preparation

**Figure 1:**
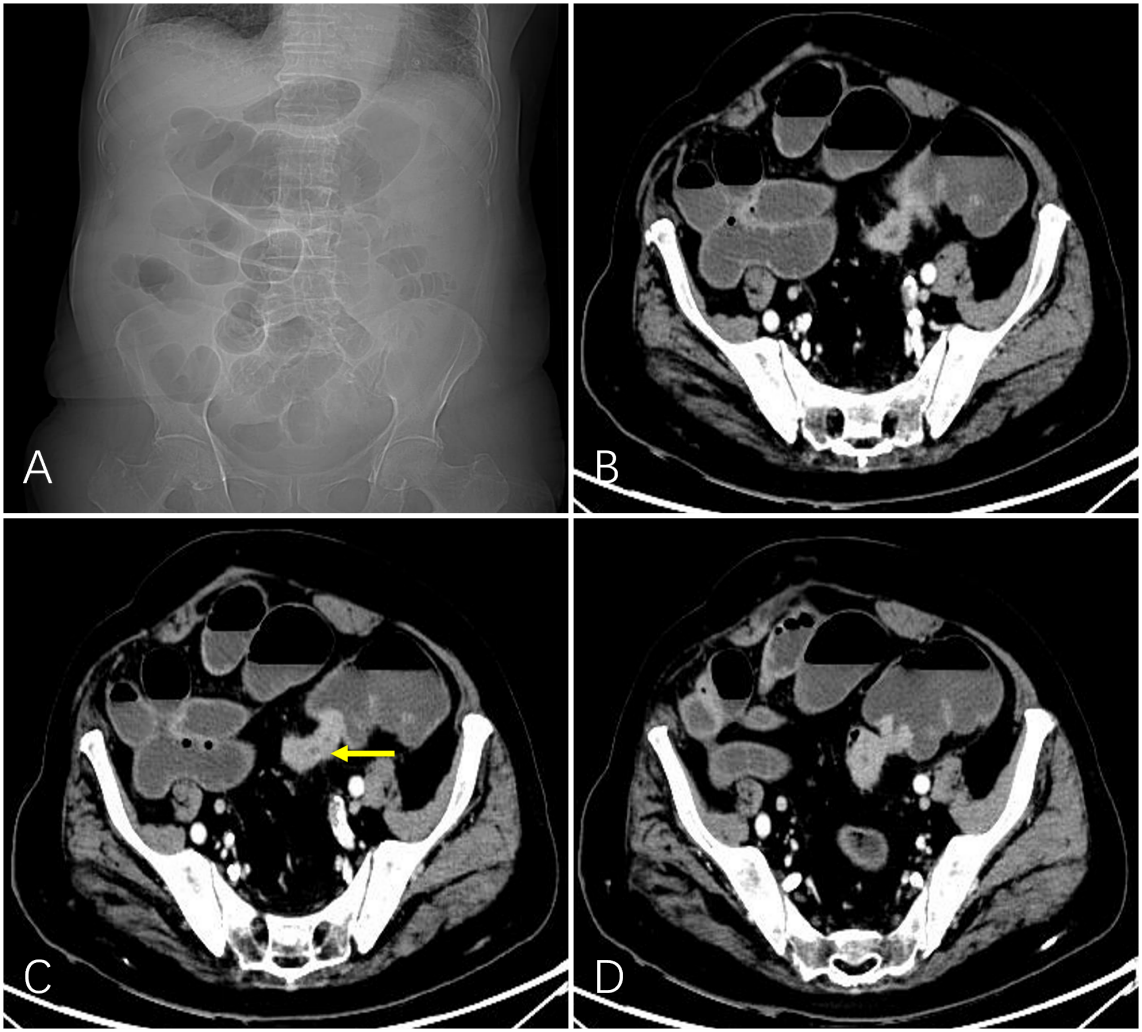
A 92-year-old man with obstructed bowel movements for 10 days and worsening for 3 days. Abdominal plain film indicated intestinal gas and fluid accumulation **(A)**. Preoperative computed tomography showed marked localized thickening of the sigmoid colon wall (indicated by the yellow arrow) and enhancement and corresponding narrowing of the intestinal lumen. The upstream intestinal tract was significantly dilated due to air and fluid accumulation **(B-D)**.

Before treatment, routine blood, liver and kidney function, electrolyte, coagulation function, and tumor marker tests; electrocardiography (ECG); and imaging (abdominal and pelvic enhanced computed tomography [CT] or magnetic resonance imaging [MRI]) were performed (**Figure 1**) to determine tumor location, the extent and degree of obstruction, tumor staging, and blood supply.

### Operation process

**Figure 2:**
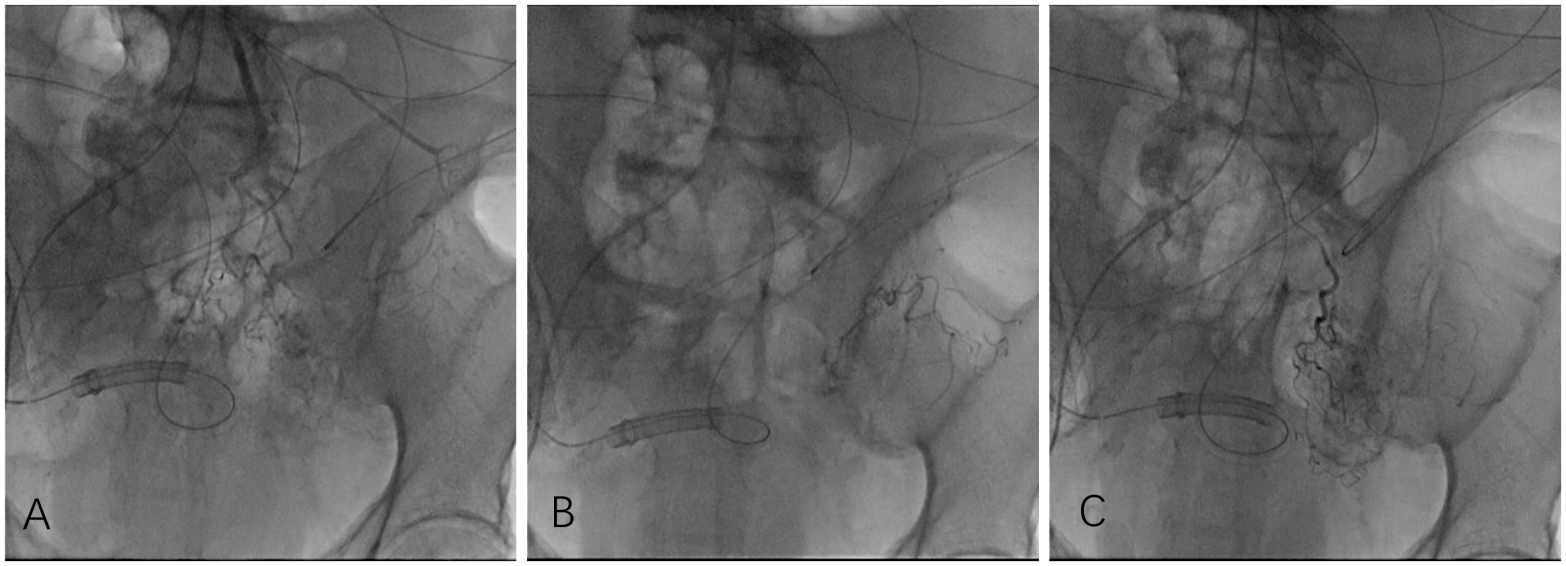
Owing to the patient’s poor breath retention, angiography was performed in digital angiography mode. Angiography showed that the inferior mesenteric artery emitted the sigmoid colon artery to participate in tumor blood supply **(A)**. After injecting chemotherapy drugs through microcatheters, chemoembolization was performed, and a total of 3 ml of iodized oil embolization emulsion was injected. Under fluoroscopy, marked deposition of iodized oil **(B, C)** was observed.

The patients lay on their backs on a digital subtraction angiography (DSA) examination bed, and a 5F vascular sheath was inserted through the right femoral artery. The 5F Cobra catheter (Cook, America) was introduced through the sheath, and inferior mesenteric artery or superior mesenteric artery angiography was performed through the catheter to visualize the tumor-feeding artery and tumor staining. Then, a 2.7F microcatheter (Terumo, Japan) was introduced into the main tumor-supplying artery through the catheter, and 50–100 mg of oxaliplatin and 2–4 mg of rituximab were sequentially injected through the microcatheter (each chemotherapy drug was configured as a 100–200 ml dilution, and the perfusion time of each drug was maintained at 15–20 min). After perfusion, the microcatheter was super-selected to the tumor-supplying arterial branch, and 20 mg of 4’-0-tetrahydropyranyladriamycin (THP) and 10 ml of lipiodol (Jiangsu Hengrui Pharmaceuticals Co., Ltd.) emulsion were slowly injected through the microcatheter under fluoroscopy for chemoembolization until the blood flow was stagnant (**Figure 2**).

### Postoperative treatment

After the operation, symptomatic treatments such as antibiotics, antiemetics, hepatoprotective drugs, acid suppression, and hydration were administered. Routine blood, liver and kidney function, and electrolyte tests were performed 3 and 7 days after the procedure. One month after the procedure, enhanced CT or MRI was performed to evaluate treatment efficacy.

### Variable definition and evaluation criteria for clinical efficacy and adverse reactions

Technical success was defined as super-selection to the tumor-supplying arterial branch and embolization to a blood stasis state. Clinical success was defined as partial or complete relief of obstruction within 1 week after interventional treatment. Clinical failure was defined as persistent unrelieved obstruction. Obstruction-free survival (OFS) was used as the primary observation indicator, defined as the time from receiving interventional treatment to the recurrence of obstruction or patient death (13). Overall survival (OS) was the secondary observation indicator.

The ColoRectal Obstruction Scoring System (CROSS) assigns a point score based on the patient’s oral intake level: CROSS 0, requiring continuous decompression; CROSS 1, no oral intake; CROSS 2, liquid or enteral nutrient intake; CROSS 3, soft solids, low residue, and full diet with symptoms of stricture; and CROSS 4, soft solids, low residue, and full diet without symptoms of stricture (14).

**Figure 3:**
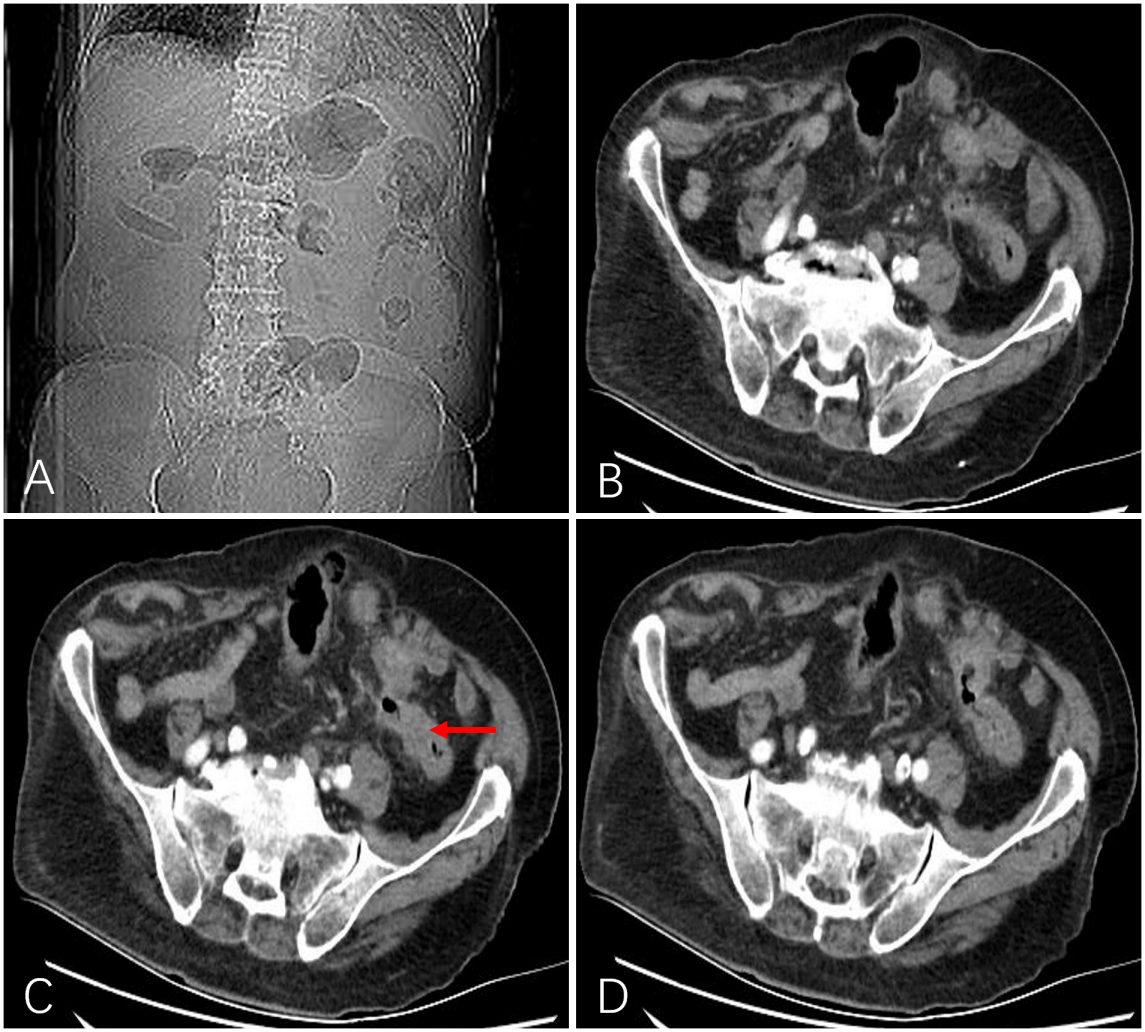
After 1 month, a follow-up abdominal X-ray showed a marked improvement in intestinal gas and fluid accumulation **(A)**. Computed tomography showed that intestinal obstruction was relieved, the lumen was unobstructed, the wall became thinner than before (indicated by the red arrow), and the enhancement was significantly weakened **(B-D)**.

According to the evaluation criteria for solid tumors (Response Evaluation Criteria in Solid Tumors version 1.1), CT or MRI examinations were performed every month to evaluate the effectiveness of primary tumor treatment (**Figure 3**). Regular follow-up observation when efficacy is assessed as CR or PRI. The patients received re-intervention when efficacy is assessed as SD or PD. Adverse events during the perioperative intervention treatment period were recorded in detail, and the patients were monitored for serious surgical complications. Adverse events were classified according to the United States Common Terminology Criteria for Adverse Events (CTCAE) General Adverse Reaction Standards.

### Follow-up

The patients were followed up through telephone or as outpatients or inpatients from the beginning of the patient receiving interventional treatment until their death or April 30, 2023. Follow-up was conducted once every month during the interventional treatment period, followed by once every 3–6 months to determine if there was recurrence of obstruction or lesion progression.

### Statistics analysis

Statistical analysis was conducted using IBM SPSS software version 26.0 (IBM, Armonk, NY). Data are presented in terms of median, mean ± standard deviation, numerical values, and percentages. The Kaplan–Meier method was used to evaluate OFS and OS.

## Results

This study included 54 patients with CRC complicated by obstruction, including 32 men and 22 women, aged 32–88 (average: 58.6 ± 14.2) years. Obstruction combined with bleeding occurred in eight cases (14.8%). In 14 cases, liver metastasis was noted. There were 28 and 26 cases with preoperative CROSS scores of 1 and 0, respectively. Patients with a CROSS score of 0 underwent intestinal obstruction catheter placement through the nose for gastrointestinal decompression. Other clinical baseline data are shown in **Table 1**.

**Table 1:** Characteristics of patients (n = 54).

	Number / Mean ± standard deviation
Sex	
Male	32 (59.3%)
Female	22 (40.7%)
Year	58.6±14.2 (32-88)
Location of the tumor	
Rectum	17 (31.5%)
Sigmoid colon	10 (18.5%)
Cecum and ascending colon	3 (5.6%)
Descending colon	8 (14.8%)
Junction of the rectum and sigmoid colon	12 (22.2%)
Transverse colon	4 (7.4%)
TNM stage	
III	32 (59.3%)
IV	22 (40.7%)
Metastasis	
Liver	14 (25.9%)
Lungs	4 (7.4%)
Omentum	2 (3.7%)
Bone	2 (3.7%)
Bladder	1 (1.9%)
Ovary	1 (1.9%)
Preoperative treatment	
Surgery	10 (18.5%)
Systemic chemotherapy	27 (50.0%)
Radiotherapy	9 (16.7%)
None	8 (14.8%)
Comorbidity	
Coronary heart disease	19 (35.2%)
Hypertension	17 (31.5%)
Diabetes	10 (18.5%)
Cerebral infarction	4 (7.4%)
Number of times interventional treatments were performed	
1	18 (33.3%)
2	34 (63.0%)
3	2 (3.7%)

All 54 patients were successfully treated with microcatheter super-selection to the tumor-supplying arterial branch and embolization to a blood stasis state (technical success rate of 100%). Angiography revealed a total of 60 arteries to be involved in tumor blood supply, with an average of 1.11 ± 0.46 branches per patient (range: 1–3 branches), including ileocolic arteries (2 cases), right colon artery (1 case), middle colon arteries (4 cases), left colon arteries (10 cases), sigmoid colon arteries (17 cases), superior rectal arteries (19 cases), inferior rectal arteries (6 cases), and median sacral artery (1 case).

All 54 patients received TAI combined with lipiodol chemotherapy embolization treatment (92 times in all). Eighteen (33.3%), 34 (63.0%), and 2 (3.7%) patients received one, two, and three treatments, respectively. Among the 36 patients who received multiple treatments, 29 patients were assessed as SD after the initial treatment, and 7 patients were assessed as PD. The time interval between multiple treatments is 1-3 months. The average lipiodol emulsion dose was 2.62 ± 1.45 ml (range: 0.5–5.5 ml). Fourteen patients (25.9%) with liver metastases underwent TACE for liver metastases.

The treatment was clinically successful in 45 (83.3%) cases. Nine patients received interventional treatment and the obstruction persisted. Five patients were subsequently implanted with intestinal stents, and four did not receive any further interventional treatment. The distribution of postoperative CROSS scores was as follows: score 0, 4 cases; score 1, 4 cases; score 2, 13 cases; score 3, 15 cases; and score 4, 18 cases. All patients with preoperatively placed intestinal obstruction catheters were successfully removed when the patient had a Cross score of ≥1. All patients experienced relief of bowel obstruction symptoms 1-8 days after surgery. No patient experienced worsening of bowel obstruction symptoms.

Fifty-four patients (100%) did not experience serious adverse events such as intestinal perforation, intestinal necrosis, or major bleeding after receiving interventional treatment. No patient experienced a significant adverse reaction during the interventional treatment process. During the follow-up period, there were no significant abnormalities in routine blood, liver and kidney function, and coagulation function tests in any patients. Common postoperative adverse reactions included gastrointestinal reactions, fever, abdominal pain, and bone marrow suppression. According to the United States CTCAE General Adverse Reaction Standards, no patient experienced grade III/IV adverse reactions (**Table 2**).

**Table 2:** Postoperative adverse reactions.

Adverse reactions	Grade /n (%)				Total
	I	II	III	IV	
Gastrointestinal reaction	14 (25.9%)	5 (9.2%)	0	0	19 (35.1%)
Fever	15 (27.8%)	1 (1.9%)	0	0	16 (29.7%)
Abdominal pain	17 (31.5%)	0	0	0	17 (31.5%)
Myelosuppression	9 (16.7%)	1 (1.9%)	0	0	10 (18.6%)
Hematochezia	6 (11.1%)	0	0	0	6 (11.1%)
Tenesmus	3 (5.6%)	0	0	0	3 (5.6%)
Mucoid stool	4 (7.4%)	0	0	0	4 (7.4%)

Evaluation of interventional treatment efficacy revealed the following results: complete response (CR), 9 cases (16.7%); partial response (PR), 28 cases (50.0%); stable disease (SD), 13 cases (22.2%); progressive disease (PD), 4 cases (11.1%); objective response rate (ORR), 66.67%; and disease control rate (DCR), 88.9%. In eight patients with concurrent bleeding, the bleeding stopped after TACE treatment, the range of time to stop bleeding is 3-7 days. Twenty-four patients subsequently received targeted immunotherapy.

**Figure 4:**
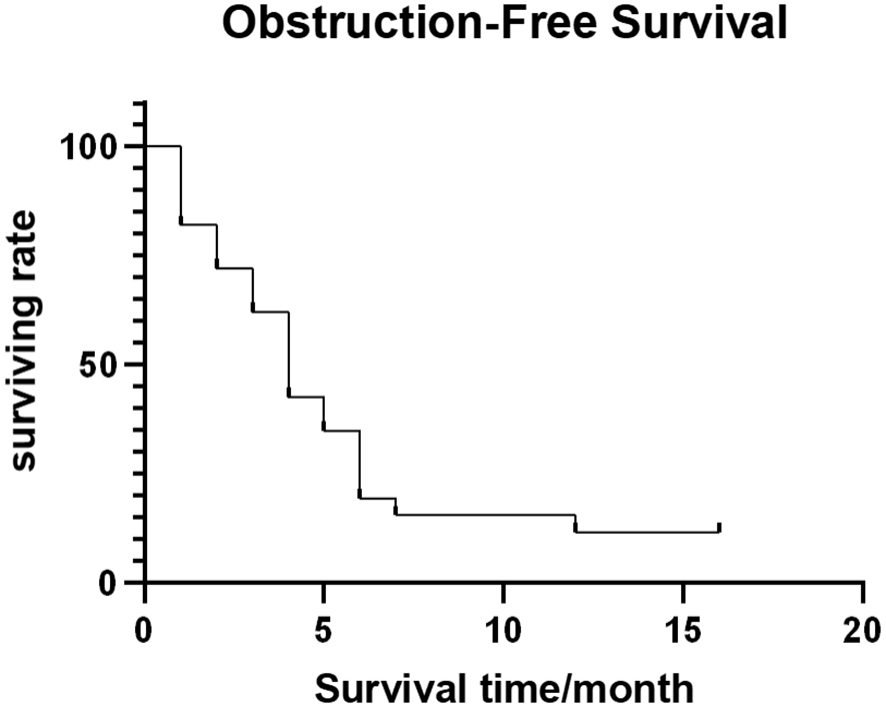
Obstruction-free survival curve.

**Figure 5:**
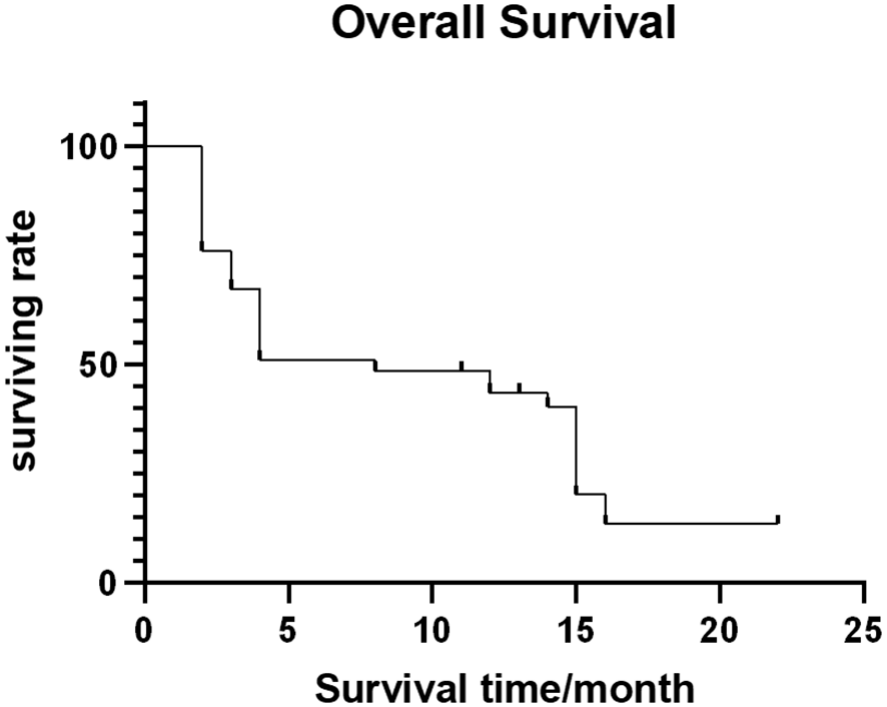
Overall survival curve.

Out of 54 patients, four were lost to follow-up. The median follow-up time was 11.5 months (range: 1-56 months). Forty-four patients (81.5%) died (28, 10, 4, and 2 patients died of multiple organ failure, lung infection and respiratory failure, gastrointestinal bleeding, and heart failure, respectively, with the average survival time being 10.5 ± 4.29 months. The median OFS of 50 successfully followed-up patients was 5.0 months (95% CI: 3.25–6.75) (**Figure 4**), and the median OS was 13.0 months (95% CI: 10.32–15.68) (**Figure 5**).

## Discussion

At present, the treatment of CRC complicated by obstruction remains challenging (15). After the formation of an obstruction, large amounts of liquid and gas accumulate in the intestinal cavity, leading to excessive dilation of the intestinal canal and edema of the intestinal wall. The toxins produced by bacterial proliferation in the intestinal cavity penetrate the blood and abdominal cavity, which can cause severe suppurative peritonitis and systemic poisoning symptoms after absorption (15, 16). Traditionally, such patients can only undergo emergency surgical procedures. However, these patients often have a poor overall condition, intestinal edema, high incidence of emergency surgical complications, and long postoperative recovery time (17). Moreover, patients with CRC complicated by obstruction tend to have an advanced tumor stage, invasion or even breakthrough of the serosa (T3/T4), and a high likelihood of positive peritoneal exfoliated cancer cells. After radical surgery, peritoneal implantation metastasis and recurrence may develop easily. Relevant literature shows that in more than 50% of patients with CRC, the opportunity for surgery is lost by the time the disease is diagnosed, or these patients may develop distant metastasis or local recurrence after surgery (18, 19). In recent years, SEMS have been widely used in clinical practice to relieve obstruction. However, the associated effective rate of stent relief is only 52.5%, and the 1-month postoperative mortality rate is 8.3%. The incidence of complications, such as stent displacement, stent restenosis and intestinal perforation, is high (20, 21).

TAI can quickly reduce tumor volume and reduce tumor burden (22, 23). Compared with traditional intravenous chemotherapy, arterial infusion chemotherapy can increase the drug titer by 2–20 times, and the therapeutic effect can be improved by 4–10 times (24), fully exerting the first-pass effect of the drug, reducing the peripheral blood drug concentration, and thus demonstrating stronger local efficacy and fewer systemic reactions (10). After perfusion chemotherapy, super-selective embolization of the target blood vessels of tumor tissue blocks the blood supply to the tumor tissue, further controlling the occurrence and development of malignant tumors, alleviating obstruction symptoms, reducing the tumor stage, and creating conditions for subsequent treatment.

In this study, lipiodol was used as a vascular embolization material for CRC. In the past, liquid embolic agents were considered contraindicated for vascular embolization treatment of hollow organ tumors. However, in this study, no serious complications such as intestinal perforation, intestinal necrosis, and massive bleeding were observed. Lipiodol, as the most commonly used embolic material at present, has good fluidity, carrier properties, and tumor affinity. It can penetrate lesions at various levels of arterial branches, leading to endothelial damage and thrombosis, cutting off of tumor-supplying arteries, and slow release of chemotherapy drugs, thereby significantly increasing the local drug concentration and action time and significantly enhancing the anticancer effect of chemotherapy drugs (25, 26). In the study of lipiodol embolization for VX2 colorectal tumors in rabbits, it was confirmed that lipiodol embolization of the colon is safe (27). The safety of lipiodol chemoembolization in the treatment of advanced CRC may be related to the following factors: 1) Lipiodol is a liquid embolic agent that can flow in blood vessels. On the one hand, it is cleared by blood flow flushing, and on the other hand, lipiodol flows back into the portal vein through capillaries and is eventually cleared by macrophages in the liver (27); 2) lipiodol is easily decomposed and absorbed by macrophages in normal tissues, thereby reducing the risk of ischemic necrosis in normal tissues; 3) super-selective embolization reduces the risk of ischemia; 4) careful control of the amount of lipiodol used and achievement of a blood stasis state under fluoroscopy; 5) lipiodol stays briefly in the mesenteric vessels and acts only as a temporary embolism. To ensure safety, it is important to note that the endpoint of embolization is a state of hemosiderosis and that no additional solid embolic particles are added.

This single-center retrospective study had a small sample. Our next step is to conduct a multicenter randomized controlled clinical trial with a large sample to verify the safety and effectiveness of TAI combined with lipiodol chemoembolization in the treatment of advanced CRC complicated by obstruction.

## Conclusion

The combination of TAI and lipiodol chemoembolization for the treatment of CRC complicated by obstruction was preliminarily proven to be safe, effective, and feasible, which is beneficial for prolonging the survival period without obstruction and improving the long-term prognosis of patients. It has broad clinical application prospects and serves as a new treatment strategy for patients with advanced CRC complicated by obstruction.

## Data availability statement

The raw data supporting the conclusions of this article will be made available by the authors, without undue reservation.

## Ethics statement

The studies involving humans were approved by The Ethics Committee of Zhengzhou University First Affiliated Hospital. The studies were conducted in accordance with the local legislation and institutional requirements. Written informed consent for participation was not required from the participants or the participants’ legal guardians/next of kin in accordance with the national legislation and institutional requirements. Written informed consent was obtained from the individual(s) for the publication of any potentially identifiable images or data included in this article.

## Author contributions

XD: Writing – original draft, Writing – review & editing. YM: Writing – review & editing. MY: Writing – review & editing. TL: Writing – review & editing. SJ: Writing – review & editing. CL: Writing – review & editing. XL: Writing – review & editing. CZ: Writing – review & editing. GZ: Writing – review & editing. GW: Writing – review & editing.
